# Draft Genome Sequences of Six Different *Staphylococcus epidermidis* Clones, Isolated Individually from Preterm Neonates Presenting with Sepsis at Edinburgh’s Royal Infirmary

**DOI:** 10.1128/genomeA.00471-15

**Published:** 2015-05-14

**Authors:** P. Walsh, M. Bekaert, J. Carroll, T. Manning, B. Kelly, A. O’Driscoll, X. Lu, C. Smith, P. Dickinson, K. Templeton, P. Ghazal, R. D. Sleator

**Affiliations:** a nSilico, Cork, Ireland; b Department of Computing, Cork Institute of Technology, Bishopstown, Cork, Ireland; c Division of Pathway Medicine, University of Edinburgh, Edinburgh, United Kingdom; d Microbiological Diagnostic Unit, Royal Infirmary, University of Edinburgh, Edinburgh, United Kingdom; e Department of Biological Sciences, Cork Institute of Technology, Bishopstown, Cork, Ireland

## Abstract

Herein, we report the draft genome sequences of six individual *Staphylococcus epidermidis* clones, cultivated from blood taken from different preterm neonatal sepsis patients at the Royal Infirmary, Edinburgh, Scotland, United Kingdom.

## GENOME ANNOUNCEMENT

*Staphylococcus epidermidis* is a Gram-positive bacterium naturally found on human skin (1) and an important opportunistic pathogen linked with neonatal blood sepsis (2–5).

Preterm neonates are a highly susceptible patient group for bacterial infections, due to their naive immune status and the invasive procedures to which they are often subjected to in neonatal ICU settings (2, 6, 7). Rapid detection of blood sepsis and characterization of the causative pathogen are critical first steps to enable appropriate treatment and improved prognostic outcomes (8–10). As part of the ClouDx-i project consortium, we aim to extend our knowledge of currently circulating pathogenic strains linked with neonatal blood sepsis to inform the continued development of new and improved molecular diagnostic assays (11). Herein, we report the draft genome sequences of six individual *Staphylococcus epidermidis* strains, isolated from preterm neonates at the Royal Infirmary, Edinburgh, United Kingdom, in 2014. Positivity for blood sepsis and species of each isolate was confirmed by classical microbiological identification and characterization techniques.

Isolates were grown overnight at 37°C on Luria broth (LB) agar, and genomic DNA was isolated using Qiagen genomic tips (Venlo, Limburg, Netherlands). Genomic DNA fragments, ranging in size from 2 to 10 kb, were generated by sonication. Fragments were subsequently used to produce a non-size-selected genome library using the Nextera mate-pair kit (Illumina, San Diego, CA). The resulting libraries were then sequenced on an Illumina MiSeq using MiSeq Reagent kit v3. Genomic sequence assembly, analysis, and automated reporting were carried out using the Simplicity software pipeline (12). The results of this analysis are summarized in Table 1. Sequence assembly was achieved using a *de-novo* assembly pipeline based on the Spades 3.10 assembly tool, with k-mers K21, K33, K55, K77, K99, and K127 nucleotides in length. Each genome was initially annotated with the Prokka tool (13) and the identified 16S rRNA genes were used to confirm the species as *S. epidermidis* in each case. Each genome was then screened using the Glimmer 3 tool (14). The predicted open reading frames (ORFs) were compared to the Uniprot Trembl database (15) using BLASTp.

**TABLE 1:** Genomic sequence assembly overview

Strain	Total no. of reads	Fold coverage	G+C content (%)	Total contigs (>1,000 bp)	Largest contig (bp)	No. of ORFs	Accession no.
NGS-ED-1107	2,599,368	135.2	32.0	53	207,733	2,570	JZUK00000000
NGS-ED-1109	2,436,309	114.6	32.0	51	360,587	2,252	JZUL00000000
NGS-ED-1110	4,476,721	223.5	32.0	45	207,733	2,515	JZUM00000000
NGS-ED-1111	5,197,793	163.5	32.0	44	250,293	2,583	JZUN00000000
NGS-ED-1117	1,607,705	76.0	32.0	48	206,303	2,459	JZUO00000000
NGS-ED-1118	1,681,207	94.1	31.9	48	206,303	2,444	JZUP00000000

Samples were handled in accordance with local ethical approval by the ethics committees of the NHS Lothian SAHSC Bioresource and NHS R&D office (project 2011/R/NE/01) and the HSS BioResource (request 13/ES/0126).

### **Nucleotide sequence accession number**s. 

This whole-genome shotgun project has been deposited at DDBJ/EMBL/GenBank under the accession numbers listed in Table 1.
